# Effect of Kruppel‐like factor 4 on PTZ‐induced acute seizure mice

**DOI:** 10.1111/jcmm.18578

**Published:** 2024-09-05

**Authors:** Bingjin Li, Jingjing Piao, Xinmiao Piao, Zihui Geng, Ziqian Cheng, Xiaohan Zou, Huiyi Jiang

**Affiliations:** ^1^ Jilin Provincial Key Laboratory on Molecular and Chemical Genetics Second Hospital of Jilin University Changchun People's Republic of China; ^2^ Department of Medical Research Centar Second Hospital of Jilin University Changchun People's Republic of China; ^3^ Department of Pediatrics The First Hospital of Jilin University Changchun People's Republic of China

**Keywords:** anticonvulsants, c‐Fos, *Klf4*, *P53*, PTZ, seizures

## Abstract

*Kruppel*‐*like factor 4* (*Klf4*) is a transcription factor that is involved in neuronal regeneration and the development of glutamatergic systems. However, it is unknown whether *Klf4* is involved in acute seizure. To investigate the potential role of *Klf4* in pentylenetetrazol (PTZ)‐induced seizure, western blotting, immunofluorescence, behaviour test and electrophysiology were conducted in this study. We found that Klf4 protein and mRNA expression were increased in both the hippocampus (HP) and prefrontal cortex (PFC) after PTZ‐induced seizure in mice. HP‐specific knockout (KO) of *Klf4* in mice decreased protein expression of Klf4 and the down‐stream *Klf4* target tumour protein 53 (TP53/P53). These molecular changes are accompanied by increased seizure latency, reduced immobility time in the forced swimming test and tail suspension test. Reduced hippocampal protein levels for synaptic proteins, including glutamate receptor 1 (GRIA1/GLUA1) and postsynaptic density protein 95 (DLG4/PSD95), were also observed after *Klf4*‐KO, while increased mRNA levels of complement proteins were observed for *complement component 1q subcomponent A (C1qa)*, *complement component 1q subcomponent B* (*C1qb*), *complement component 1q subcomponent C* (*C1qc*), *complement component 3* (*C3*), *complement component 4A* (*C4a*) and *complement component 4B* (*C4b*). Moreover, c‐Fos expression induced by PTZ was reduced by hippocampal conditional *KO* of *Klf4*. Electrophysiology showed that PTZ‐induced action potential frequency was decreased by overexpression of *Klf4*. In conclusion, these findings suggest that *Klf4* plays an important role in regulating PTZ‐induced seizures and therefore constitutes a new molecular target that should be explored for the development of antiepileptic drugs.

## 1 INTRODUCTION

Seizure disorders are common, chronic and serious neurological disorders throughout the world.^1^ Although there are many therapeutic methods to treat seizure disorders, they are not effective in many patients, and some patients are completely resistant to all current treatments. Moreover, most anticonvulsants present a variety of side effects that limit the effective use in many patients. Additionally, these conditions often present early in life and early exposure to anticonvulsants may be associated with the increased risk of developing psychiatric disorders, including autism spectrum disorder.^2^ Some anticonvulsants may also worsen other psychiatric symptoms, inducing manic symptoms.^3^, ^4^ Consequently, the development of new molecular targets for antiepileptic drugs is a critical need in the treatment of seizure disorders.

It is well known that *Klf4* induces pluripotency in stem cells (*iPSCs*).^5^ *Klf4*, a zinc finger‐containing transcription factor, has been reported to play roles in stem cell differentiation,^6^ cancer^7^, ^8^ and atherosclerosis.^9^ *Klf4* is highly expressed in the cerebral cortex, hippocampus (HP) and hypothalamus^10^, ^11^ and has important developmental roles in regulating glutamatergic function.^12^ In the central nervous system (CNS), *Klf4* has been implicated in neuron regeneration,^13^, ^14^ brain tumour formation,^15^ neuronal apoptosis,^16^ and the pathophysiology of traumatic brain injury^17^ and Alzheimer's disease.^18^, ^19^ Given the apparent broad role of *Klf4* in brain plasticity^20^ and neurodegenerative disorders, it might also be thought that *Klf4* would influence general brain excitability and therefore have a role in seizure disorders. Nonetheless, our understanding of the involvement of *Klf4* in seizure disorders remains unclear.

*P53* is a downstream mediator of *Klf4* actions that are important in amyloid β‐protein^19^ or Traumatic brain injury (TBI)‐induced neuroinflammation.^21^ Whether a result of this neuroinflammatory cascade or other actions of *Klf4*, changes in brain excitability involving *p53* are unclear. Activated *p53* occurs in pilocarpine‐induced drug resistant epilepsy,^22^ pentylenetetrazol (PTZ)‐induced seizures^23^ and post‐traumatic epilepsy (PTE) animal brains.^24^ PTZ‐induced seizures in rats.^23^ This likely results from increased neuronal activity as the neuronal marker (NeuN) c‐Fos is also increased by PTZ, while anticonvulsant treatments decrease this elevation in c‐Fos expression.^16^, ^25^, ^26^ HP is an important brain region related to seizures. c‐Fos expression is increased by convulsants, and decreased by anticonvulsants, in the HP and PFC.^25^ In this study, we investigated the anticonvulsant effect of *Klf4* in PTZ‐induced acute seizures in mice, along with behavioural and molecular characterization of the effect of conditional hippocampal *Klf4*‐KO on PTZ‐induced seizures. The effect of overexpression of the *Klf4* on PTZ‐treated hippocampal neuronal excitability was also determined using whole‐cell current‐clamp recordings.

## 2 RESULTS

### 2.1 Expression of KLF4 after PTZ‐induced acute seizure in mice brain

**FIGURE 1:**
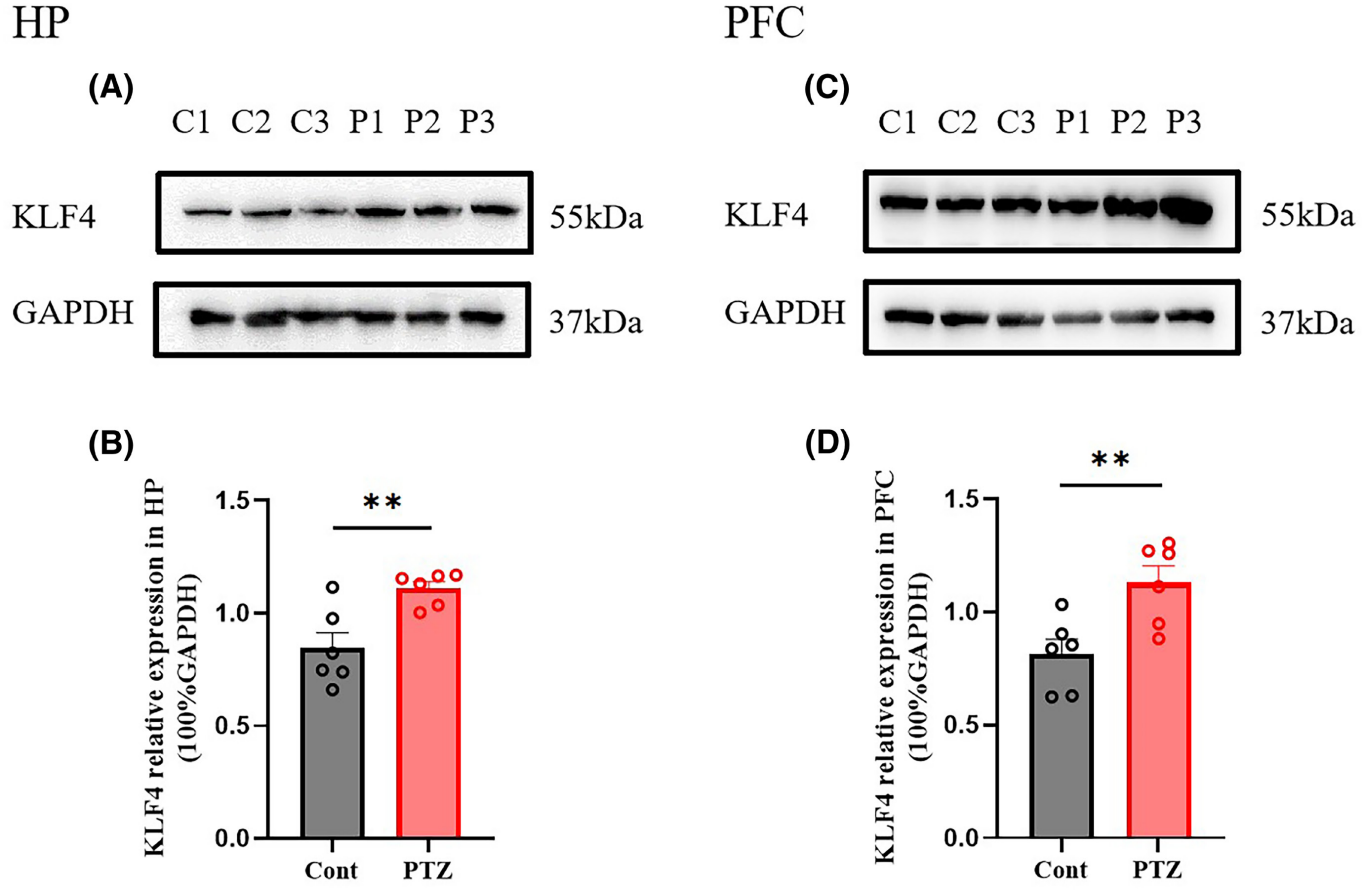
Protein expression levels of transcription factors in in acute PTZ‐induced seizure. (A, B) HP Klf4 protein expression in the acute PTZ‐treated mice; (C, D) FC Klf4 protein expression in the acute PTZ‐treated mice. PTZ, Pentylenetetrazole (70 mg/kg, ip). Columns represent the mean ± S.E.M. *n* = 6. **p < 0.01 versus PTZ group.

**FIGURE 2:**
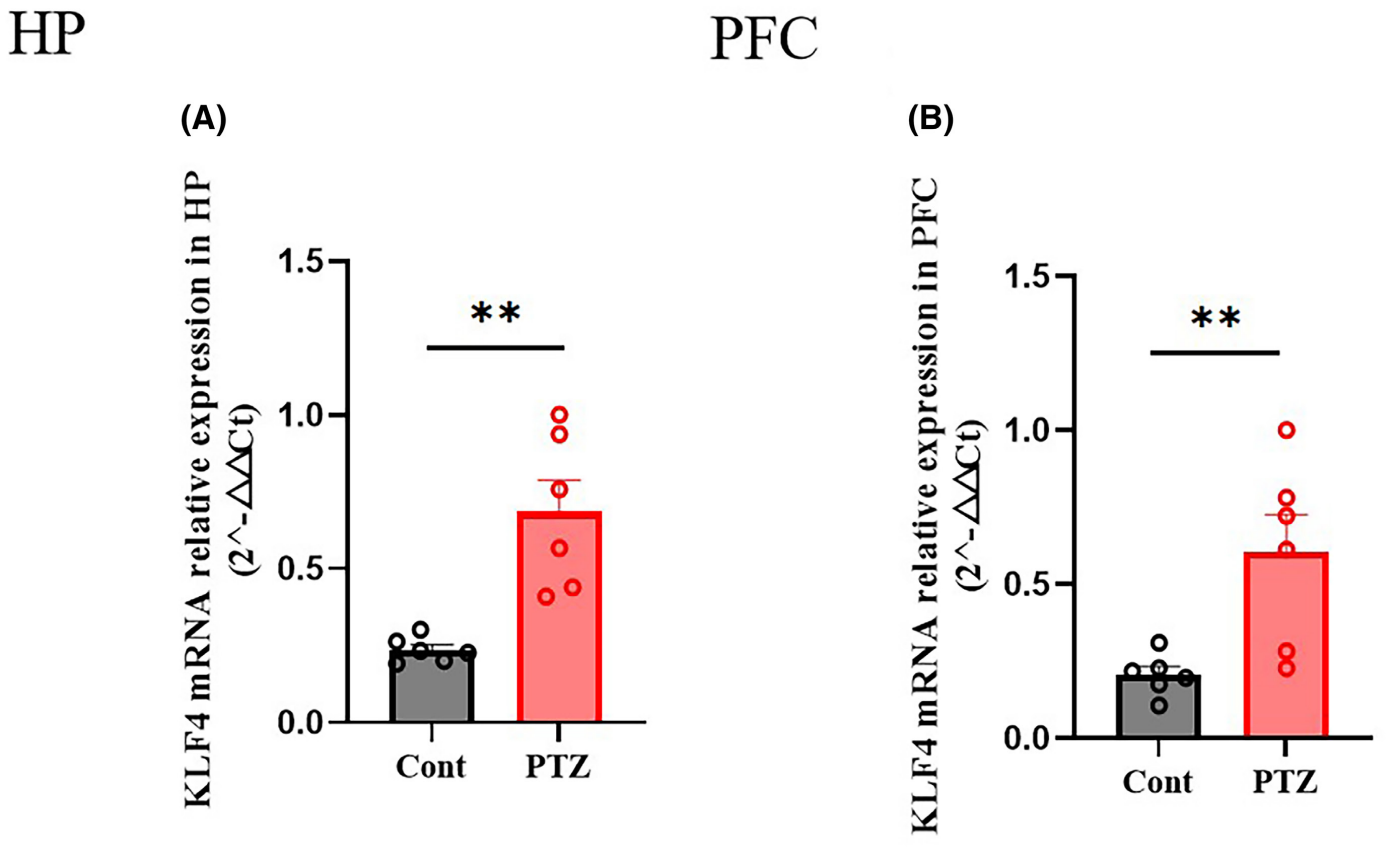
Brain mRNA expression of transcription factors in acute PTZ‐induced seizure. (A) HP *Klf4* mRNA expression level in the acute PTZ‐treated mice; (B) FC *Klf4* mRNA expression in the acute PTZ‐treated mice. PTZ, Pentylenetetrazole (70 mg/kg, ip). Columns represent the mean ± S.E.M. *n* = 5–6. **p < 0.01 versus PTZ group.

Figures 1 and 2 show hippocampal protein expression levels of *Klf4* after PTZ‐induced acute seizure. Klf4 protein expression was significantly increased after a single PTZ administration both in HP (Figure 1A,B: *p* < 0.01) and PFC (Figure 1C,D: *p* < 0.01) of mice. PCR results for *Klf4* mRNA expression are shown in Figure 2. *Klf4* mRNA expression was also significantly increased after single PTZ administration both in HP (Figure 2A: *p* < 0.01) and PFC (Figure 2B: *p* < 0.01) of mice.

### 2.2 Behaviour characterization of conditional hippocampal knockout of *Klf4* in mice

**FIGURE 3:**
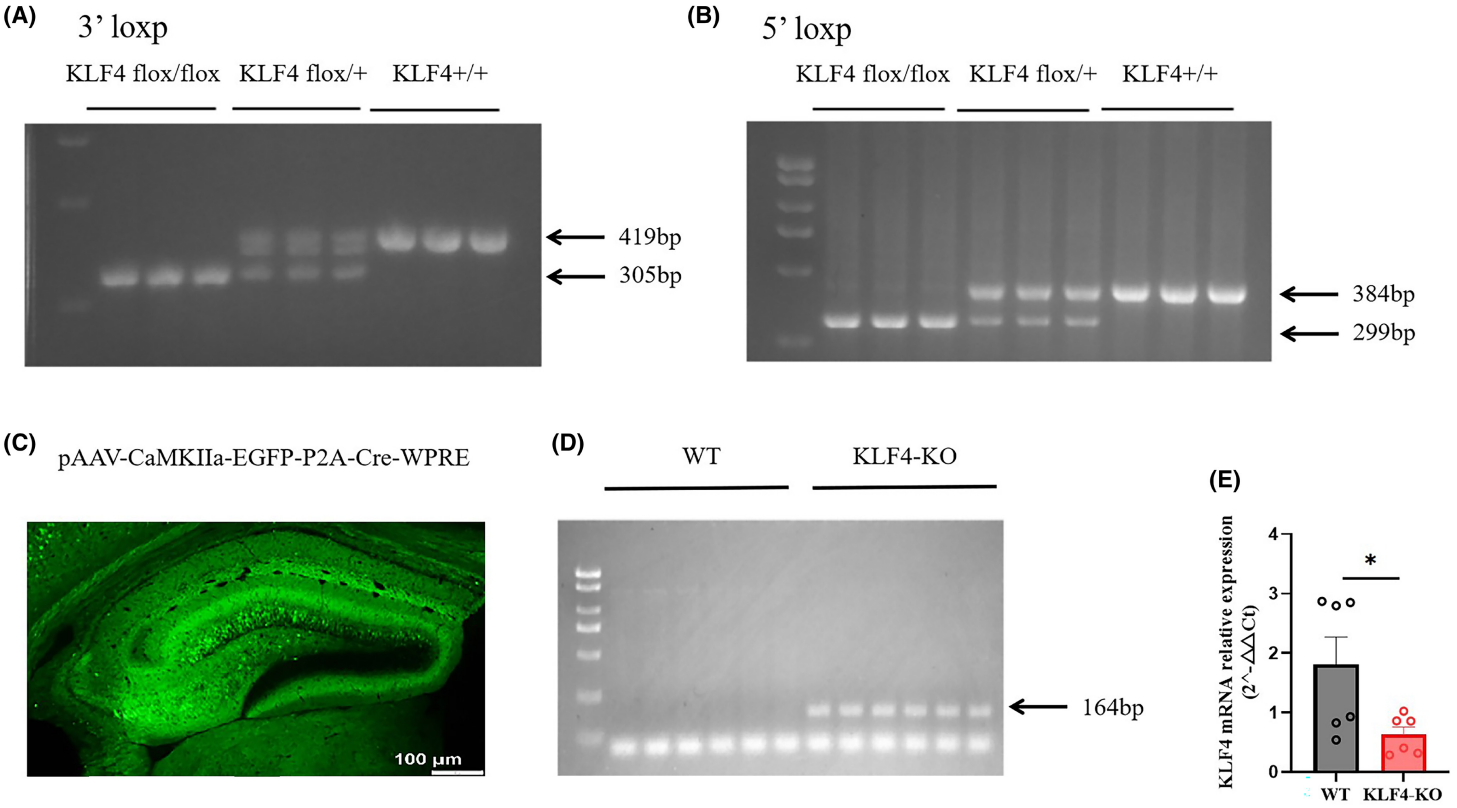
Generation of conditional hippocampal *Klf4‐*KO mice. (A) Genotyping results of 3′loxp *Klf4* +/+ mice; (B) genotyping results of 5'loxp *Klf4*+/+ mice; (C) HP injection of virus pAAV‐CaMKIIa‐EGFP‐P2A‐Cre‐WPRE; (D) genotyping results of conditional hippocampal *Klf4‐*KO mice; (E) MRNA identification of conditional hippocampal *Klf4‐*KO mice. Columns represent the mean ± S.E.M. *n* = 6. *p < 0.05.

**FIGURE 4:**
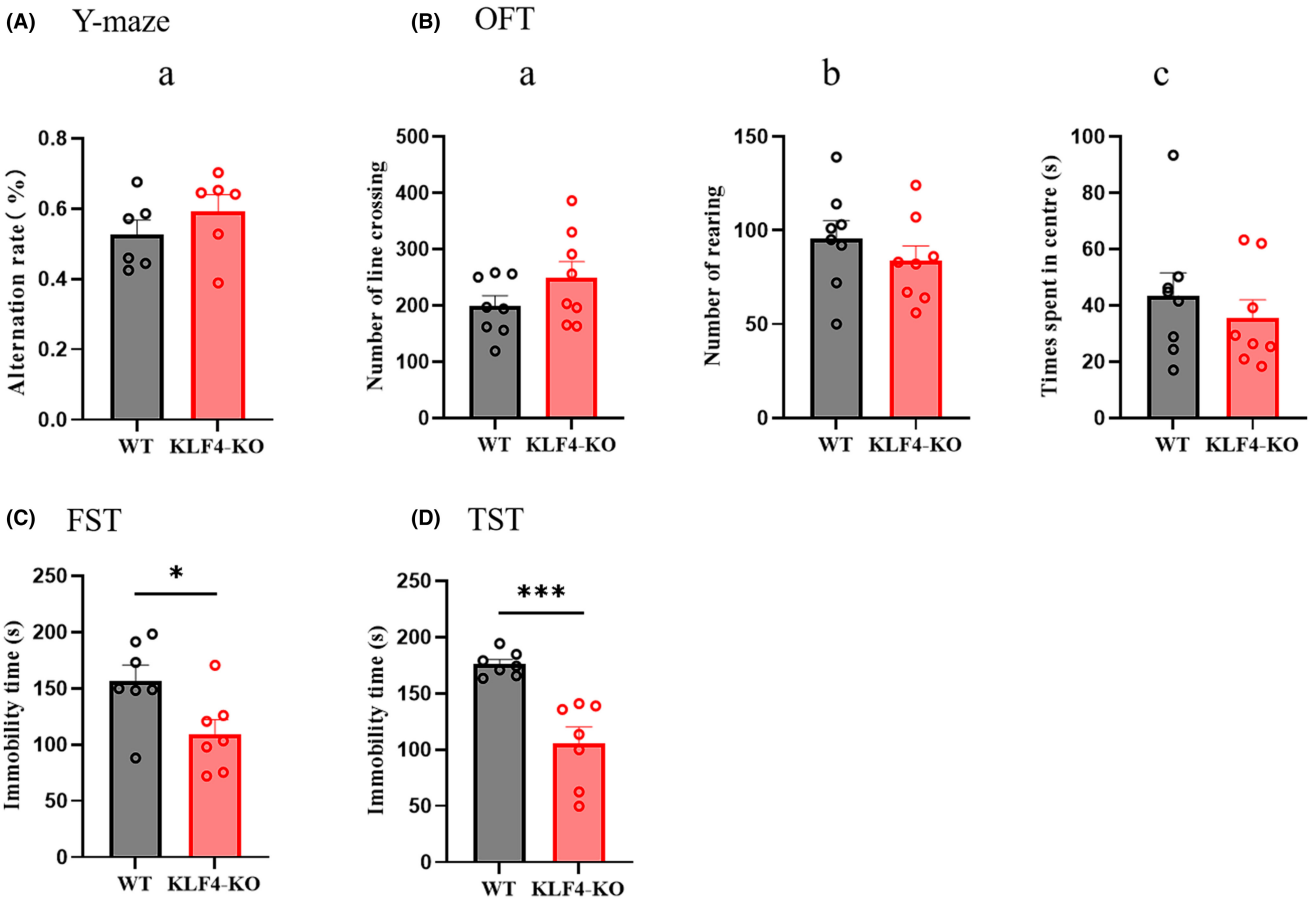
Behaviour characterization of conditional hippocampal *Klf4‐*KO mice. (A) Alteration rate in Y‐maze test; (B) number of line crossings in open field test; (C) number of rears in open field test; (D) time spent in the centre of an open field. Columns represent the mean ± S.E.M. *n* = 6. *p < 0.05 and ***p < 0.001 versus WT.

Figure 3 shows genotyping results for conditional hippocampal knockout (KO) of *Klf4* in mice. Figure 3A,B show the genotype identification of Elovl4 floxed mice. Figure 3C shows the hippocampal injection site of the pAAV‐CaMKIIa‐EGFP‐P2A‐Cre‐WPRE. PCR results show decreased hippocampal mRNA expression in conditional hippocampal *Klf4*‐KO mice (Figure 3D,E). Figure 4 shows behavioural results in conditional hippocampal *Klf4*‐KO mice compared to wild‐type mice. There were no significant behavioural differences between wild‐type and conditional hippocampal knockout of *Klf4*‐KO mice in the Y‐maze test and open field test (OFT). The only behavioural differences were in the forced swimming test (FST) and tail suspension test (TST), where immobility time was significantly decreased after conditional hippocampal *Klf4*‐KO (Figure 4C FST: *p* < 0.05; Figure 4D TST: *p* < 0.01).

### 2.3 Molecular characterization of conditional hippocampal *Klf4*‐KO mice

**FIGURE 5:**
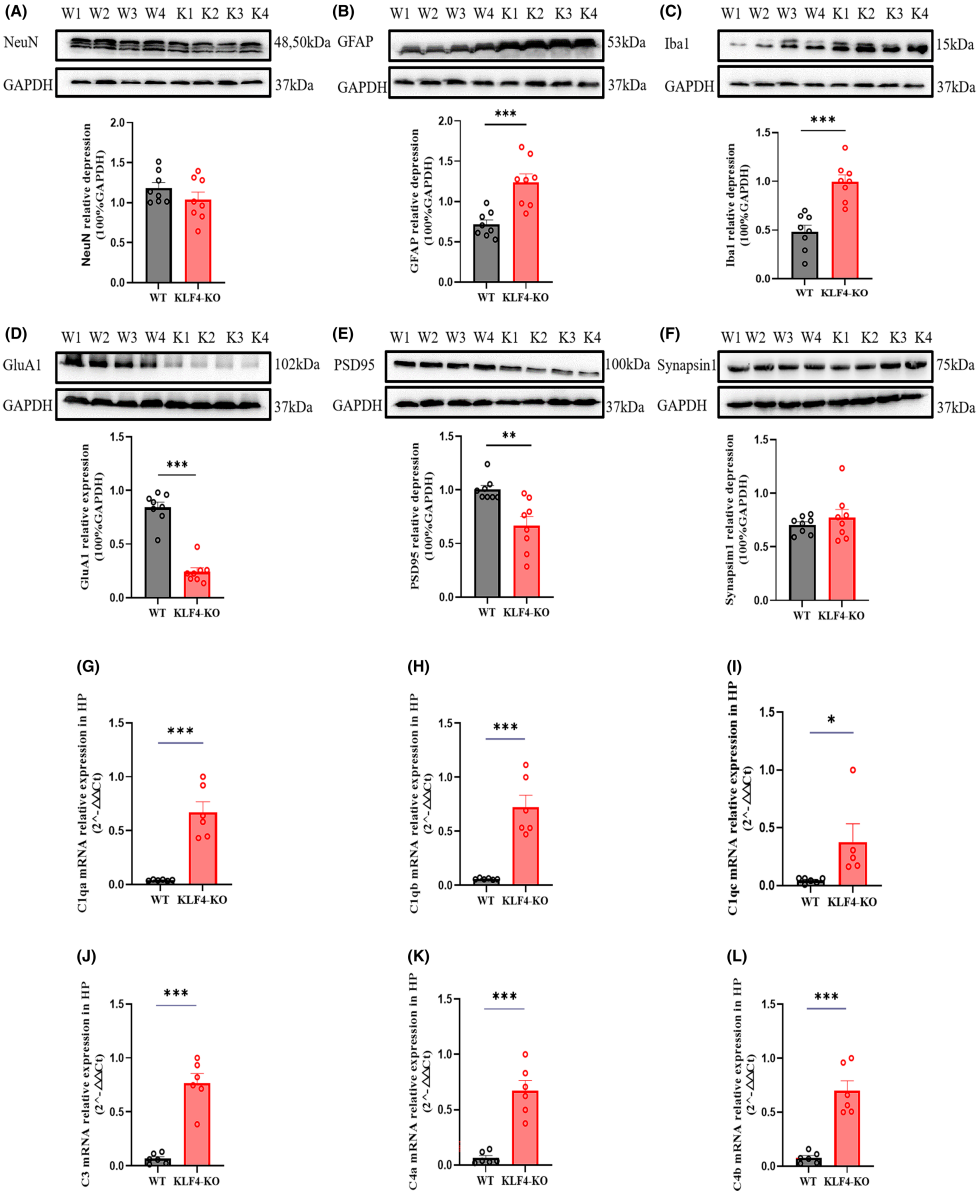
Molecular characterization of conditional hippocampal *Klf4*‐KOmice. (A) HP NeuN protein expression; (B) HP GFAP protein expression; (C) HP Iba1 protein expression; (D) HP GluA1 protein expression; (E) HP PSD95 protein expression; (F) HP Synapsin1 protein expression; (G–L), mRNA level of HP complement (*C1*: *C1qa*, *C1qb* and *C1qc*; *C4*: *C4a* and *C4b*) expressions. Columns represent the mean ± S.E.M. *n* = 6–8. *p < 0.05, **p < 0.01, ***p < 0.001 versus WT.

Figure 5A–F shows the molecular changes after conditional hippocampal *Klf4‐*KO in the HP of mice using a NeuN, a marker of astrocytes (GFAP), a microglial marker (Iba1), and markers for synaptic proteins (GluA1, PSD95 and Synapsin1) comparing wild‐type and conditional hippocampal *Klf4‐*KO using western blot. *Klf4*‐KO mice had increased protein expression of GFAP (*p* < 0.001) and Iba1 (*p* < 0.001) compared to wild‐type mice. Decreased expression of the synaptic proteins GluA1 (*p* < 0.001) and PSD95 (*p* < 0.01) were observed. There were no differences in the expression of NeuN or synapsin 1. Figure 5G–L shows complement changes between WT and *Klf4‐*KO mice by PCR. mRNA levels of all complement proteins were very low in WT mice and substantially and significantly increased after conditional hippocampal *Klf4*‐KO (*c1qa* and *c1qb*, *p* < 0.001, Figure 5G,H; *c1qc*, *p* < 0.05, Figure 5I; *c3*, *c4a* and *c4b*, *p* < 0.001, Figure 5J–L).

### 2.4 Effects of conditional hippocampal *Klf4‐* KO on PTZ‐induced acute seizure and the expression of *Klf4*, *P53* and c‐Fos

**FIGURE 6:**
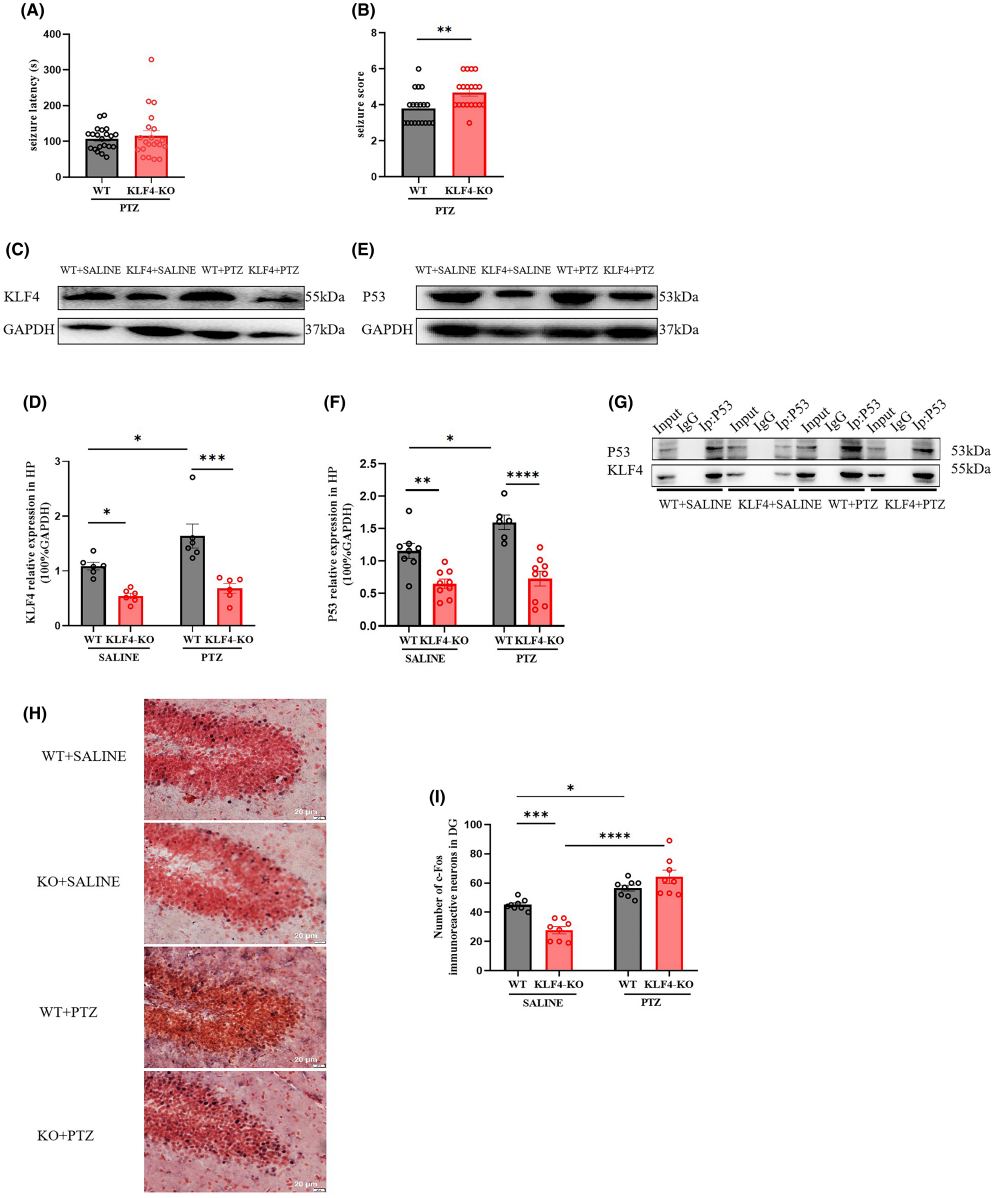
Effects of conditional hippocampal *Klf4‐*KO on PTZ‐induced acute seizure. (A, B) Seizure latency and score; (C, D) hippocampal protein expression of Klf4; (E, F) hippocampal protein expression of p53; (G) co‐immunoprecipitation of Klf4 and P53; (H, I) The expression of c‐Fos was assessed in the dentate gyrus (DG) region of the hippocampus. Columns represent the mean ± S.E.M. *n* = 6–8. *p < 0.05, **p < 0.01, and ***p < 0.001.

As shown in Figure 6, conditional hippocampal *Klf4‐*KO increased the PTZ‐induced seizure score (Figure 6B, *p* < 0.01), but had no effect on seizure latency (Figure 6A). Figure 6C,D shows the results of western blotting examining the expression of Klf4 and its downstream target protein, P53. Conditional hippocampal *Klf4‐*KO decreased protein levels of Klf4 in both PTZ and saline treatment groups (two‐way ANOVA, genotype: *F*(1, 20) = 7.401, *p* < 0.05; PTZ treatment: *F*(1, 20) = 35.13, *p* < 0.0001, Figure 6C,D). Post hoc Tukey comparisons showed that PTZ increased Klf4 protein expression (*p* < 0.05), while *Klf4‐*KO reduced Klf4 protein levels *p* < 0.001, protein levels of p53 were also decreased by conditional hippocampal *Klf4‐*KO in both saline and PTZ‐treated mice (two‐way ANOVA, genotype: *F*(1, 28) = 6.146; PTZ treatment; *F*(1, 28) = 43.16, post hoc Tukey test, *p* < 0.01, Figure 6E,F). Post hoc Tukey comparisons showed that PTZ increased p53 protein expression (*p* < 0.05), the effect was reversed by conditional hippocampal *Klf4‐*KO (*p* < 0.01). To explore the effect of PTZ on the interaction between proteins in the HP, co‐immunoprecipitation was performed. Figure 6G shows co‐immunoprecipitation results for Klf4 and p53. There was a direct interaction between *Klf4* and *p53* in the HP, which was increased by PTZ. Figure 6H,I shows c‐Fos expression in the dentate gyrus of the HP. There were significant differences across treatment groups in the dentate gyrus of HP (PTZ treatment and genotype interaction *F*(1, 36) = 6.583, *p* < 0.05; PTZ treatment, *F*(1, 36) = 69.53; *p* < 0.0001; genotype, *F*(1, 36) = 17.38, *p* < 0.0001, Figure 6H,I). PTZ treatment increased c‐Fos expression slightly in the HP of control mice and *Klf4‐*KO mice (control mice: *p* < 0.00001; *Klf4‐*KO mice: *p* < 0.00001). *Klf4‐*KO decreased PTZ‐induced c‐Fos expression in the dentate gyrus of the HP (*p* < 0.01).

### 2.5 Effects of overexpression of *Klf4* on action potential frequency of hippocampal pyramidal neurons

**FIGURE 7:**
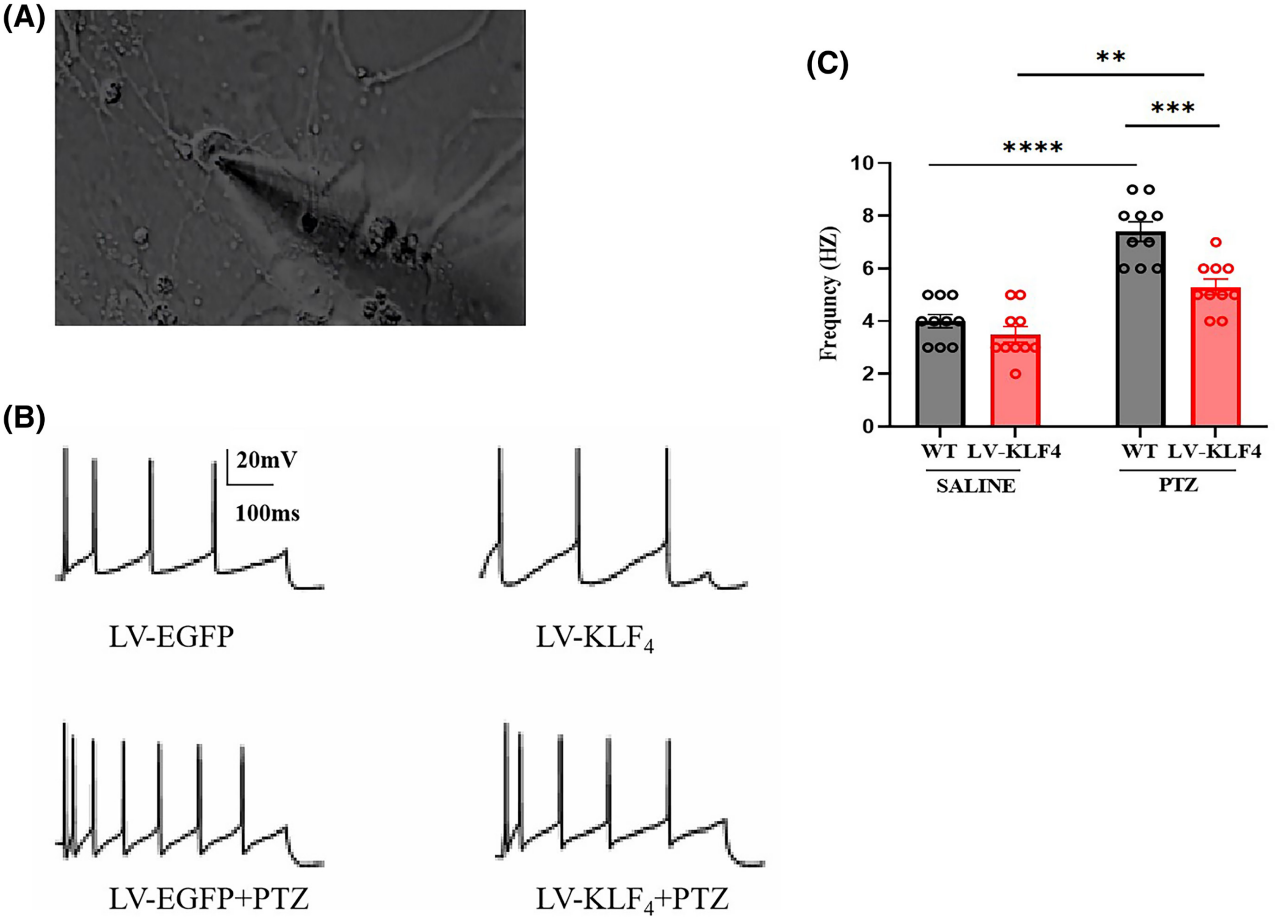
Effects of overexpression of *Klf4* on action potential (AP) frequency in hippocampal pyramidal neurons. (A) Visualization of the primary cultured hippocampal neuron. (B) The representative recording shows that action potentials were inhibited by *Klf4* overexpression. (C) The mean frequency plots showing changes in AP frequency between groups. Columns represent the mean ± S.E.M. *n* = 10. *p < 0.05, **p < 0.01, and ***p < 0.001.

As Figure 7A–C shows that viral over‐expression of *Klf4* alone had no effect on action potential (AP) frequency. PTZ increased AP frequency in cells from control (LV‐EGFP) mice (two‐way ANOVA: F (3, 36) = 31.166, *p* < 0.001; *p* < 0.001 vs. saline) and this effect was reduced by overexpression of *Klf4* (*p* < 0.01 vs. LV‐EGFP mice treated with PTZ). There was no significant difference between saline‐treated LV‐EGFP cells and PTZ‐treated LV‐*Klf4* treated cells on AP frequency.

## 3 DISCUSSION

This study revealed that the transcription factor *Klf4* was regulated following PTZ‐induced acute seizures in mice. Glutamatergic stimulation induces swift upregulation of *Klf4* expression in cultured neurons and *Klf4* overexpression activated caspase‐3 after treatment with NMDA (10 μM).^12^ Consistent with these overall roles, the present experiments demonstrate that *Klf4* plays a protective role in the acute seizure. In addition, viral‐mediated over‐expression of *Klf4* gene expression in vitro also increased seizure latency. Thus, both approaches are consistent with the role of *Klf4* in reducing seizure susceptibility.

To further explore the role of the *Klf4* in the epilepsy, conditional hippocampal *Klf4*‐KO mice were constructed, and behaviour and molecular characterization were performed. *Klf4*‐KO mice did not affect the behaviours in the Y‐maze and OFT. There was a selective effect upon depressive‐like behaviour where *Klf4*‐KO mice exhibited reduced immobility time in the FST and TST. The reason may be related to changes in neural excitability in *Klf4‐*KO mice under some conditions. Repeated electroconvulsive therapy decreases immobility time in the FST,^27^, ^28^ so that a similar change in hippocampal excitability may occur here. Although there were no differences at baseline, PTZ‐induced seizure activity was reduced. Supporting this relationship between depressive phenotypes and hippocampal excitability, in a genetic absence epilepsy model in Wistar Albino Glaxo/Rijswijk (WAG/Rij) rats decreases in immobility time in the FST are observed.^29^

Protein expression levels of neuronal and glial markers including *NeuN*, *GFAP* and *Iba1* in HP were detected through western blotting. *GFAP* and *Iba1* were increased in the *Klf4*‐KO mice. Activated glia also produce cytokines, produce neuronal hyperexcitability and inflammation that may contribute to the pathogenesis of epilepsy.^30^, ^31^ A recent study reported that overexpression of *Klf4* confers vascular protection via reducing cerebral vascular endothelial inflammation.^32^ Although some evidence has shown that *Klf4* plays a critical role in CNS function and susceptibility to some neurological disorders, the specific mechanisms are still obscure. Importantly, *Klf4* contributes to microglia‐mediated neuroinflammation in Alzheimer's disease.^19^, ^33^ However, another study reported that LPS stimulation increased *Klf4* expression in microglial cells in a time and dose‐dependent manner. *Klf4* resulted in decreased levels of the pro‐inflammatory cytokines such as *TNF‐α*, *MCP‐1*, *IL‐6* and *IFN‐gamma*.^34^, ^35^ The apparent inconsistency of these results may relate to different roles of *Klf4* under different conditions. Further work needs to be done to show if changes in *Klf4* are a cause or result of some of these inflammatory changes.

In the present study, synaptic proteins including GluA1, PSD95 and Synapsin1 were also detected by western blotting in the *Klf4*‐KO mice. GluA1 and PSD95 were decreased in PTZ‐treated mice, as in a previous study.^36^ That study reported that ICV injection of the four reprogramming factors *Oct4*, *Sox2 c‐MYC* and *Klf4* increases protein expression of PSD95, thereby reversing brain damage‐induced synapse plasticity.^37^ These results indicated that *Klf4* may play an important role in synaptic plasticity. Protein levels of synapsin are not changed after *Klf4*‐KO. Similar to what was observed here, activated microglia and complement cascade *C1q* signalling in the HP may contribute to synaptic loss in a mouse model of neuroinflammation induced by repeated LPS injections.^38^ Inflammation also decreases PSD95 in the HP.^39^ Moreover, complement mRNA expression was also tested by PCR here. These complement factors are mediators of inflammation, and also involved in the molecular mechanisms of epilepsy.^40^ In this study complement *1q* (*a*, *b* and *c*), *1q3* and *1q4* (*a* and *b*) all were increased in *Klf4*‐KO mice. The complement *C3‐C3aR* pathway mediates microglia‐astrocyte interactions following status epilepticus (SE)^41^, ^42^ reported that *C4B* (but not *C3*)‐deficient mice exhibited increased susceptibility to seizures induced by PTZ and failed to upregulate the expression of multiple immediate early genes (IEGs) including *Egrs1‐4* and c‐Fos. These findings suggest that seizure behaviour may be related to inflammation‐related microglial and astrocytic activation and further affects synaptic protein changes.

In this study, conditional hippocampal *Klf4*‐KO did not affect seizure latency. Viral‐mediated overexpression of *Klf4* in vitro did increase seizure latency. This may have to do with differences in circumstances. An acute seizure may increase Klf4 protein expression,^43^ but chronic seizure may decrease Klf4 protein expression. Decreased Klf4 protein levels were found in a PTZ‐kindling mouse model of epilepsy.^20^ In addition, in a small sample size clinical study expression of *Oct4*, *Sox2*, *c‐MYC* and *Klf4* genes was increased after initial electroconvulsive stimulation. Gene expression levels after treatment were significantly different from the initial gene expression.^44^ Our recent work found that *Klf4* can exert sedative effects in pentobarbital‐treated mice through modulation of p53 and the Stat3 pathway in the hypothalamus.^45^ Further study is needed to evaluate potential biphasic changes in *Klf4* expression in the development of the seizure.

The western blotting study indicated that PTZ increased *Klf4* expression, and the effect was reversed by *Klf4*‐KO. Further examination of a potential downstream mediator of these effects showed that *p53* was also increased by PTZ, and the effect was reversed by *Klf4*‐KO. These results are consistent with a previous study which found that *P53* was increased by PTZ treatment.^23^ *P53* as a downstream mediator of *Klf4* is well studied in inhibition of proliferation and tumour suppression.^46^, ^47^ Elevated *P53* levels have been reported in epilepsy animal models and the hippocampuis of epilespsy patients.^22^, ^48^ In the present study, *Klf4*‐KO regulated the Klf4‐p53 pathway and increased seizure scores. Recently, some evidence showed a regulatory role of *p53* in neuronal activity. Deletion of *p53* protects neurons from stress and damage.^49^, ^50^ It was also found that depletion of *p53* attenuated cocaine‐induced kindling behaviours and associated c‐Fos immunoreactivity in the HP.^51^ In addition, consistent with this hypothesis, we found that *Klf4*‐KO mice had increased p53 protein levels after PTZ treatment and PTZ increased interactions between Klf4 and p53 in the HP. These findings align with the results that the downregulation of p53 induced by *Klf4* overexpression attenuated PTZ‐induced excessive neuronal activities.

To a certain extent, all of these effects involve alterations in brain excitability, which were consistent with both the immunohistochemistry and electrophysiology experiments. In the c‐Fos immunofluorescence study, in the DG of the HP and piriform cortex, PTZ was associated with increases in c‐Fos positive neurons. These effects were reversed by overexpression of *Klf4*. Consistent with the above findings, these results indicate that alterations in neuronal activity (and neuronal excitability) in the HP may contribute to the anticonvulsant effects of *Klf4* overexpression. These results are consistent with a previous report showing that PTZ affects the HP,^25^ and that *Klf4* is involved in the inhibition of c‐Fos expression in the regions. Consistent with the Western blotting and immunohistochemical studies, the electrophysiological study found that overexpression of *Klf4* reverses the increases in AP frequency in hippocampal pyramidal cells produced by PTZ.

Reprogramming factors (*Klf4*) might be potential molecular targets for neurogenesis. Febrile seizures or SE may lead to transient upregulation of adult neurogenesis in the HP^52^, ^53^ and may have a role in later epileptogenesis. Moreover, acute PTZ‐induced generalized tonic–clonic seizures were increased the number of proliferating cells in both the dentate gyrus and the subventricular zone. Therefore, increased *Klf4* after a single PTZ administration may be related to neurogenesis and neuroprotection. In terms of its relationship to chronic seizure, *Klf4* protein expression and neurogenesis may involve more complex mechanisms. Further study is needed to evaluate the detailed mechanisms.

In summary, the results of this study suggest that *Klf4* is increased in PTZ‐induced seizures, and the mechanisms Klf4‐p53 pathway may be involved in the observed behavioural outcomes. Inhibition of this pathway by hippocampal *Klf4‐*KO exhibits proconvulsant effects. Similarly, overexpression of *Klf4* in vitro showed anticonvulsant effects as shown by reduced action potential frequencies. These findings suggest that *Klf4* may constitute a novel molecular target for the development of anticonvulsive agents. However, this study has some limitations that should be addressed in future research. First, the effects of *Klf4* manipulation were only examined in the HP. Considering the potential impact of *Klf4* in different neuronal populations or brain regions, future studies should explore its role in other areas of the brain. Additionally, investigating the function of *Klf4* in other seizure models, such as genetic models or chronic epilepsy models, could provide a broader understanding of its role in epilepsy pathophysiology. The specific molecular mechanisms of the *Klf4* anticonvulsant effects need to be further investigated, particularly under more chronic circumstances.

## 4 MATERIALS AND METHODS

### 4.1 Animals

Imprinting Control Region (ICR) strain male ICR mice (age: 6–8 weeks) were purchased from Jilin University (Changchun, China). Animals were housed in plastic cages (mice: 25.5 × 15 × 14 cm) and maintained in standard laboratory conditions (temperature: 23 ± 1°C, lights on and off: 07:00 and 19:00), with ad libitum access to food and water. All experiments were carried out in accordance with the Guide for Animal Experimentation of Jilin University and every effort was made to minimize any potential suffering experienced by the animals.

### 4.2 Drugs

PTZ was purchased from Sigma‐Aldrich (St. Louis, MO, USA). The drug was dissolved in sterile, pyrogen‐free saline. Doses of PTZ (70 mg/kg) were selected based on previous studies.^7^, ^25^

### 4.3 Experimental design

**FIGURE 8:**
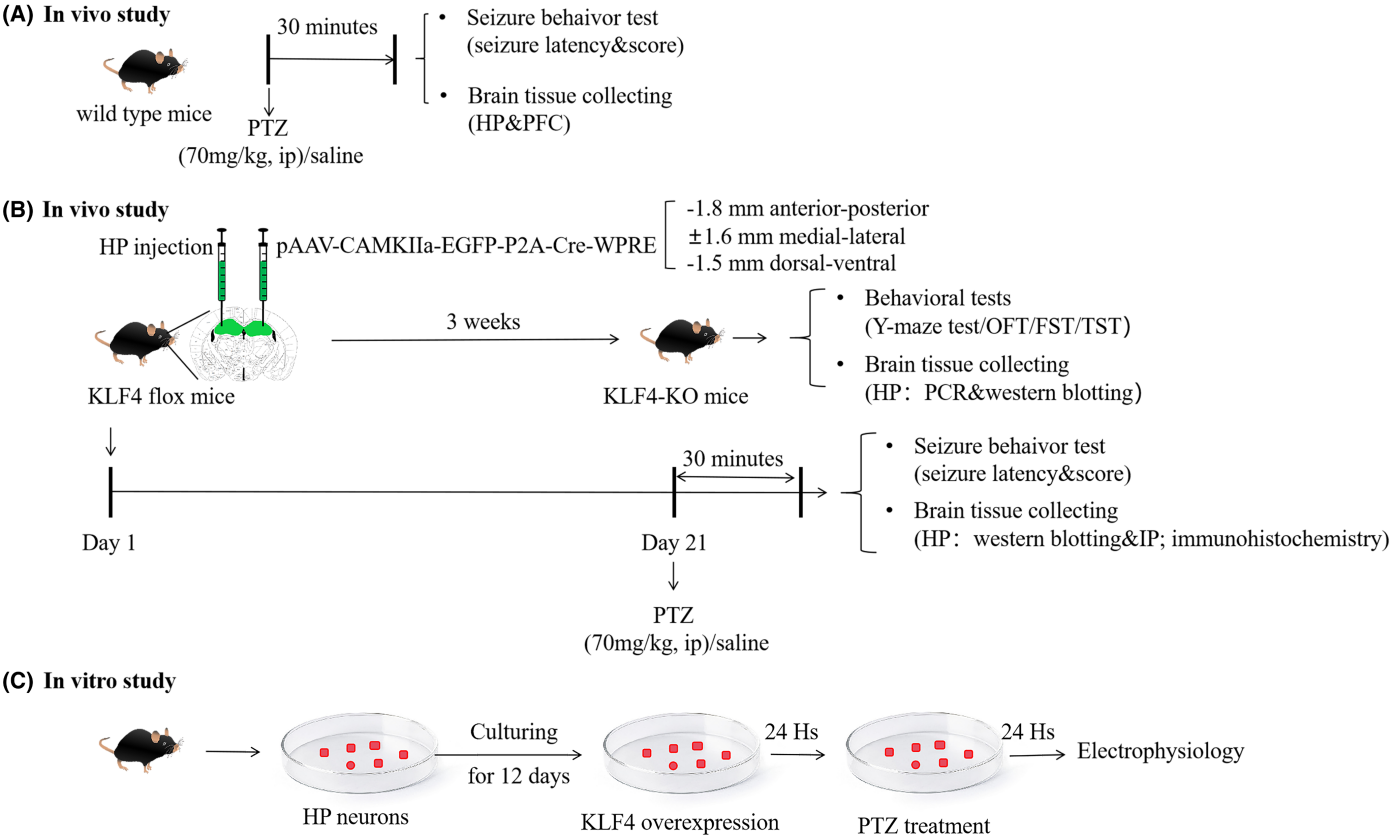
Experimental design and procedures. Experimental design and procedures. (A) In vivo study: Behavioral assessment following single PTZ injection and collection of brain tissue. (B) In vivo study: upper: Behavior and molecular characterization of conditional hippocampal *Klf4*‐KO mice; lower: The effect of conditional hippocampal *Klf4*‐KO on PTZ‐induced acute seizure mice behavior and brain tissue collection. (C) In vitro study: Electrophysiological recordings from hippocampal neurons.

To investigate the effects of PTZ on *Klf4* in mice and its related mechanisms, mice were divided into two groups: a control group (*n* = 6) and PTZ group (*n* = 6). To investigate the behaviour and molecular characterization of conditional hippocampal KO of *Klf4* in mice, mice were divided into two groups: control mice (behaviour test: *n* = 6–8; molecular test: *n* = 6–9) and *Klf4*‐KO mice (behaviour test: *n* = 6–8; molecular test: *n* = 6–9). To examine the effects of conditional hippocampal *Klf4*‐KO on PTZ‐treated mice, mice were divided four groups: WT + saline (*n* = 8), WT + *Klf4*‐KO (*n* = 9), PTZ + WT (*n* = 6) and PTZ + *Klf4*‐KO (*n* = 9). Behavioural tests were conducted immediately after the onset of seizures. The mice were monitored for 30 min post‐seizure induction, and samples for molecular analysis were collected immediately after the behavioural tests. In addition, the effects of *Klf4* overexpression on PTZ‐treated neurons, four groups: WT + saline (*n* = 10), LV‐*Klf4* + saline (*n* = 10), PTZ + WT (*n* = 10) and PTZ + LV‐*Klf4* (*n* = 10) were used in in vitro study. Figure 8 outlines the timeline and procedures for the experiments.

### 4.4 Seizure latency after PTZ injection

Behavioural changes in the test subjects were monitored for 30 min after PTZ injection (65 mg/kg, ip). Seizure behaviour was categorized following the classification method outlined in our previous study^54^: Mice were taken from their home cages and placed individually into Plexiglas cages (25.5 × 15 × 14 cm) after PTZ injection and observed for 30 min. PTZ seizure scale: Stage 0, characterized by no response; Stage 1, exhibiting eating behaviour and facial twitching; Stage 2, displaying myoclonic body jerks; Stage 3, demonstrating forelimb clonus and rearing behaviour; Stage 4, experiencing clonic convulsions and turning onto the side; and Stage 5, showing generalized clonic convulsions and loss of righting. Additionally, the latencies for the onset of myoclonic jerks and clonic seizures were measured, with a maximum observation period of 30 min following the PTZ injection.^54^ Mice were survived 30 min after PTZ injection.

### 4.5 Behaviour study

#### 4.5.1 Y‐maze test

Y‐maze test was performed following a previously outlined protocol.^55^ In summary, individual mice were introduced to one of the arms and given 8 min for unrestricted exploration. The alternation score (%) was computed using the following formula [(number of consecutive sets of three arm choices, each including all three arms)/(total arm entriesa−2)] × 100. An arm entry was recorded when all four limbs of the mouse were inside an arm.

#### 4.5.2 Open field test

OFT was employed to assess locomotor and exploratory behaviours. Mice were individually placed in a black acrylic cylinder (diameter: 48.8 cm; height: 16 cm) divided into 19 equal squares by black lines. The horizontal and vertical locomotor activities of mice were recorded using a camera for a 6‐min duration. Each animal was tested only once. Following the trial, the chamber was cleaned to avoid any impact on the results of subsequent experiments. Detailed experimental procedures can be found in previous reports.^56^, ^57^

#### 4.5.3 Forced swimming test

FST was carried out following the previously described method.^58^ Mice were placed in a cylindrical transparent plastic tank with a diameter of 11 cm and a depth of 25 cm, filled with water to a depth of approximately 10 cm, and maintained at a temperature of 25 ± 1°C. After a 2‐min adaptation period, the observation was initiated in an environment free from external disturbances. The immobility time of mice were observed during the last 4 min. The FST was conducted in a quiet environment with no significant changes in lighting conditions. After each trial, mice were removed from the water and returned to their cages, and the water in the tank was replaced.

#### 4.5.4 Tail suspension test

TST was also carried out following the previously described method.^59^ The posterior one‐third of the tail of each mouse was taped and suspended approximately 40 cm above the ground for 6 min. Immobility time during the last 4 min was measured. Immobility was defined as a lack of active escape movements and maintaining a vertical position during suspension. To prevent mutual interference between mice during the experiment, opaque black partitions were used to separate individual mice, ensuring they did not come into contact with each other.

### 4.6 *Klf4* conditional hippocampal KO mice and *Klf4* overexpression

Conditional hippocampal *Klf4*‐KO mice were generated from Klf4 flox+ mice by hippocampal virus injection. *Klf4* flox+ mice were constructed based on CRISPR/Cas9 by Biocytogen Pharmaceuticals (Beijing) Co., Ltd. All mice (6–8 weeks) shared the same C57/BL6J genetic background (Jackson Laboratory stock). *Klf4* flox/flox mice were obtained by crossing *Klf4* flox+ mice. Conditional hippocampal *Klf4*‐KO mice were generated by injection of pAAV‐CAMKIIa‐EGFP‐P2A‐Cre‐WPRE virus in the HP (AP: −2 mm; ML: +1.8 mm, DV: −2 mm) of *Klf4* flox/flox mice. Viral‐mediated gene transfer of a *Klf4* vector to over‐express *Klf4* in the dorsal HP was studied using a lentivirus expression vector synthesized by Obio Technology (Shanghai, P.R. China). *Klf4* was also constructed to a pLenti‐Ubc‐EGFP‐2A‐MCS‐3FLAG vector. Packages of pLenti viruses were provided by Obio Technology, Shanghai, China. Viral titers were 1×10E8 particles/mL for pLenti‐Ubc‐EGFP‐2A‐*Klf4*‐3FLAG, 1×10E9 particles/mL for pLenti‐Ubc‐EGFP‐2A‐MCS‐3FLAG‐control. Stereotaxic intra‐hippocampal injection was performed as described. Mice were deeply anaesthetised by intraperitoneal injection of pentobarbital (65 mg/kg). A volume of 0.5 μL pLenti‐Ubc‐EGFP‐2A‐*Klf4*‐3FLAG or pLenti‐Ubc‐EGFP‐2A‐MCS‐3FLAG was infused through a glass pipette (0.2 μL/min) bilaterally in the dorsal HP (1.5 mm ventral to skull surface, ±1.6 mm from the midline, and −1.8 mm anterior to bregma). The pipette was left in place for a minimum of 5 min following the injection to prevent backflow. Subsequent to the lentivirus injection, all animals were housed in a standard environment for 3 weeks for recovery before experiments began.

### 4.7 RNA extraction, reverse transcription PCR and real‐time PCR

Each mouse brain tissue (HP or PFC) sample was placed in 1 mL TRIzon reagent (CWBIO, China) following the manufacturer's instructions. cDNA synthesis was conducted according to the instructions of the reverse transcription kit (GenStar, China). Quantitative real‐time PCR was performed according to the instructions provided with the qPCR kit, utilizing the Genious 2X SYBR Green Fast qPCR Mix (Abclonal, China). GAPDH was used as an internal control. The primers used in qPCR were synthesized by Beijing Genomics Institution.

Primers used in qPCR were as follows:

*Klf4*‐forward sequence (5′‐TACACTGAGTCCCGAGGAACT‐3′).

*Klf4*‐reserve sequence (5′‐TACACTGAGTCCCGAGGAACT‐3′).

*GAPDH*‐forward sequence (5′‐AATGTGTCCGTCGTGGATCTG‐3′).

*GAPDH*‐reserve sequence (5′‐AGCCCAAGATGCCCTTCAGTG‐3′).

Primers used in genotyping were as follows:

3LoxP‐forward sequence (5′‐ATGGTTCCCATTGCAGGGATTCTGG‐3′).

3LoxP‐reserve sequence (5′‐AGTTGGCTCTGTTGAAACCCAAAGGA‐3′).

5'LoxP‐forward sequence (5′‐TGGGCGCACTCAGTCCACTATATT‐3′).

5LoxP‐reserve sequence (5′‐GGAACTGGACAGAACAGGAAGGCAG‐3′).

*Klf4*‐forward sequence (5′‐GAAGGGAGAAGACACTGCGTCCAG‐3′).

*Klf4*‐reserve sequence (5′‐TGTCACACTTCTGGCACTGAAAGGG‐3′).

### 4.8 Co‐immunoprecipitation

Brain tissue collection and protein extraction were the same as western blotting. Co‐immunoprecipitation was quantified using a Co‐IP kit (abs995, Absin, Shanghai, China) according to the manufacturer's instructions. Initially, 5 μL of Protein A and 5 μL of Protein G were integrated into the 500 μL sample and incubated at 4°C for 60 min, followed by centrifugation at 12,000*g* for 1 min at 4°C. One microgram of primary antibody (p53, mouse monoclonal antibody, ab26, Abcam, Shanghai, China; or Mouse IgG, A7028, Beyotime; as appropriate) was added to the supernatant and incubated overnight at 4°C with gentle mixing. The samples were then washed with 500 μL wash buffer (1×) and centrifuged at 12,000*g* for 1 min. Forty microlitres of SDS sample buffer (1×) was added to the precipitate, and the samples were added and incubated in a water bath at 95–100°C for 5 min. Followed by centrifugation at 14,000*g* for 1‐min, primary antibodies p53 (1:800, ab26, mouse monoclonal antibody, Abcam) and Klf4 (1:1000, ab129473, rabbit polyclonal antibody, Abcam, Shanghai, China) were added.

### 4.9 Western blotting for Klf4 and p53

Fresh bilateral whole HP was removed 30 min after PTZ administration. The tissue was homogenized by gently crushing the tissue 20–30 times in a glass tissue grinder in lysis buffer (137 mM NaCl, 20 mM TRIS, 1% NP40, 10% glycerol, 1 mM phenylmethylsulfonyl fluoride (PMSF), 10 μg/mL aprotinin, 1 μg/mL leupeptin, 0.5 mM sodiumvanadate, and 0.5 mM sodium flouride) on ice. The homogenate was centrifuged at 10,000 rpm for 20 min at 4°C. Protein extracts were separated on 10% SDS‐PAGE gels and transferred to polyvinylidene difluoride membranes. Membranes were incubated overnight in Tris Buffered Saline/0.1% Tween‐20 with anti‐Klf4 (ab72543; 1:600; Abcam), p53 (sc6243;1:1000; Santa Cruz), and beta‐actin (ZB2301;1:8000). After multiple washes with Tris‐buffered saline containing Tween 20, the membranes were incubated with the respective peroxidase‐labelled secondary antibody (goat anti‐rabbit IgG‐HRP ZB‐2301; 1:500 for Klf4, 1:1500 for p53 and 1:5000 for beta‐actin; ZSGB‐BIO).

### 4.10 C‐Fos immunofluorescence

Two hours after the PTZ injection, mice were deeply anaesthetised with 65 mg/kg pentobarbital and perfused transcardially with 0.1 M phosphate buffered saline (PBS), followed by 4% paraformaldehyde in PB (pH 7.4). The brains were carefully extracted from the skull and post‐fixed with 4% paraformaldehyde for 24 h, followed by 30% sucrose in PBS for 3 days. The HP was sectioned into 15‐μm coronal slices with a cryostat (Leica CM1860; Leica Microsystems, Germany). The sections were rinsed in PBS and then blocked by 10% goat serum for 60 min and then rinsed in 0.1 M PBS. Primary c‐Fos antibodies (sc253; 1:1000; Santa Cruz Biotechnology) were diluted to 1:1000 and incubated overnight at 4°C on a rotating shaker. Following the three washes with 0.1 M PBS, the sections were incubated in Alexa Fluor 594‐conjugated goat anti‐rabbit secondary antibody (A11012; 1:1000; Zhongshan) for 1 h at room temperature. Subsequently, the slices were imaged using an Olympus microscope (BX51).

### 4.11 Electrophysiology

#### 4.11.1 Cell culture

Primary HP neurons were cultured from embryonic Day 17–18 C57BL/6J mice on plates coated with poly‐L‐lysine (Sigma; molecular weight 300,000). The neurons were maintained in Neurobasal A media (Invitrogen) supplemented with B27 (Invitrogen), Glutamax™ (Thermo Fisher Scientific) and Antibiotic‐AntiMYCotic (Thermo Fisher Scientific). Hippocampal neurons were cultured for 12 days, and then the constructed *Klf4* overexpression vectors were transfected for 24 h. The neurons were treated with 10 mM PTZ for 24 h, and then cells were used for electrophysiological recordings.

#### 4.11.2 Patch clamp recordings

Neuronal membrane potentials were assessed via whole‐cell current‐clamp recordings by using a MultiClamp 700B amplifier (Molecular Devices, USA). A culture dish containing cells was positioned on the stage of an inverted microscope (BX51WI; Olympus, Japan), while patch pipettes, filled with an intracellular solution comprising 130 K‐gluconate, 20 KCl, 10 HEPES, 4 Mg‐ATP, 0.5 Na3‐GTP and 0.2 EGTA. The pH was adjusted to 7.3 with 10 M KOH and the osmolarity was set between 280 and 300 mOsm. Resistance of the pipette ranged between 3 and 5 MΩ. Recordings were performed only when the series resistance was below 20 MΩ. To enhance the quality of recording from pyramidal neurons, phase‐bright cells with noticeably large, pyramidal soma were preferentially selected. Cultured neurons featuring modest dendritic arborizations, elongated axons, and soma diameters ranging from 15 to 25 μm were *chosen* for electrophysiological assessments. Monitoring of whole‐cell resistance and resting membrane potential were used as an index, ensuring stability in these parameters during the experiment. Data acquisition and analysis were facilitated using Clamp‐fit 10.3 software (Axon, USA).

#### 4.11.3 Statistical analyses

All data are expressed as mean ± SEM. Statistical analyses were conducted using SPSS Statistics for Windows, Version 20.0 (Armonk, NY, USA). Differences between the experimental groups were compared using one‐way or two‐way analysis of variance (ANOVA) with factor 1 (PTZ treatment) and factor 2 treatment (*Klf4*‐KO or overexpression), and Tukey's HSD post hoc test for comparisons between means. A significance level of *p* < 0.05 was considered statistically significant.

## AUTHOR CONTRIBUTIONS

**Bingjin Li:** Conceptualization (equal); investigation (equal); writing – original draft (equal); writing – review and editing (equal). **Jingjing Piao:** Methodology (equal); writing – review and editing (equal). **Xinmiao Piao:** Data curation (equal); formal analysis (equal); methodology (equal); project administration (equal). **Zihui Geng:** Data curation (equal); formal analysis (equal); methodology (equal). **Ziqian Cheng:** Supervision (equal); validation (equal); visualization (equal). **Xiaohan Zou:** Methodology (equal); resources (equal); software (equal). **Huiyi Jiang:** Funding acquisition (equal); investigation (equal); writing – review and editing (equal).

## FUNDING INFORMATION

This work was supported by NSFC [The National Natural Science Foundation of China, grant number 82371540], Project of Jilin University Education and Teaching Reform & Research (No. 2022JGY021), Department of Science and Technology of Jilin Province (No. 20200201504JC). Thank you so much for Song Qin for providing valuable comments on the construction of *Klf4*‐KO mice.

## CONFLICT OF INTEREST STATEMENT

Not applicable.

## Data Availability

Not applicable.
